# Editorial: Eating disorders as a challenge for public health: from health policies to evidence-based treatments

**DOI:** 10.3389/fpubh.2025.1662260

**Published:** 2025-07-25

**Authors:** Octavian Vasiliu, Paolo Meneguzzo, Adela Magdalena Ciobanu, Mara Carsote

**Affiliations:** ^1^Clinical Neurosciences Department, “Carol Davila” University of Medicine and Pharmacy, Bucharest, Romania; ^2^Department of Psychiatry, “Dr. Carol Davila” University Emergency Central Military Hospital, Bucharest, Romania; ^3^Department of Neuroscience, University of Padua, Padua, Italy; ^4^Department of Psychiatry, “Prof. Dr. Al. Obregia” Clinical Hospital of Psychiatry, Bucharest, Romania; ^5^Department of Endocrinology, “C.I. Parhon” National Institute of Endocrinology, Bucharest, Romania; ^6^Department of Endocrinology, “Carol Davila” University of Medicine and Pharmacy, Bucharest, Romania

**Keywords:** eating disorders, anorexia nervosa, bulimia nervosa, binge eating disorder, orthorexia nervosa, public health policies, COVID-19, food neophobia

## 1 Introduction

Eating disorders (EDs) represent a challenge for public health because of their negative impact on individuals' overall functionality, healthcare costs, caregivers' burden, high rate of comorbidities, all of these being reflected in synthetic indicators like global disability-adjusted life-year (DALY) and quality-adjusted life year (QALY) (1, 2). As reflected in the data from the Global Burden of Disease 2021, the global age-standardized rates for DALYs, incidence, and prevalence of EDs in adolescents and young adults increased significantly from 1990 to 2021, with values of 76.51, 380.96, and 357.10, respectively, per 100,000 individuals in 2021 (2). Additionally, the modeled projections are not encouraging, as by 2040, the worldwide prevalence of ED is expected to increase up to 373.48 per 100,000 individuals (2). This, however, is only part of the more complete image of the mental health-related burden, with depressive disorders, schizophrenia, neurocognitive disorders, autism spectrum disorders, substance use disorders, and other mental disorders being placed among the 25 leading causes of years lived with disability (YLD) (3, 4). Therefore, the need for early detection and treatment of ED and comorbid disorders is urgent, and requires health policies targeting these objectives, in order to increase the chances of functional recovery, especially in adolescents and young adults, who are the most affected by these disorders.

Secondly, it is noteworthy that the current state of research on EDs can be considered paradoxical. This is because, although numerous epidemiological and clinical studies have been dedicated to the prevalence and risk factors for this pathology, data regarding evidence-based treatments for EDs are scarce, and the efficacy of such interventions is

modest (5). This could come as hardly a surprise, since, for example, only 0.4% of the mental health research expenditure in the UK is dedicated to EDs (6).

Thirdly, the existing information about health policies dedicated to early detection, prophylaxis, and treatment of EDs is highly heterogeneous and inconsistent. The stigma associated with EDs should also be targeted by such policies, since it may represent an important shortcoming in the addressability of patients, early diagnosis, and adequate monitoring of EDs (7).

Finally, this urgency became even more apparent during the COVID-19 pandemic, which disrupted access to care and intensified emotional distress in vulnerable populations, patients diagnosed with EDs included (8). Therefore, the need for redesigning treatment pathways to ensure resilience during public health crises is granted based on the existing, preliminary, evidence (8).

**Figure 1:**
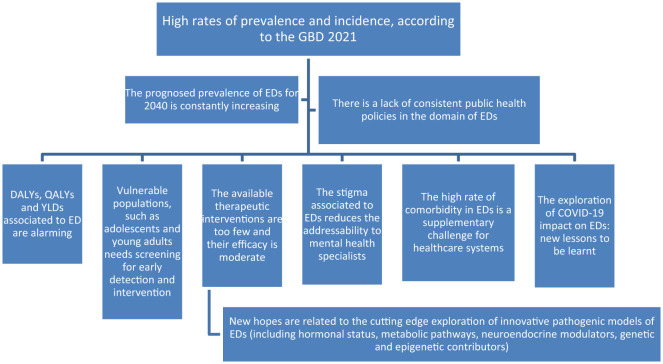
Eating disorders as a global health challenge. EDs, eating disorders; GBD, Global Burden Disease report; DALYs, disability-adjusted life-years; QALYs, quality-adjusted life years; YLDs, years lived with disability.

For all these reasons, represented in Figure 1, this Research Topic of articles was intended to collect new research in the field of EDs, with the aim of aiding the development of public health policies.

## 2 Emerging areas of research in the field of eating disorders as a public health challenge

Gao et al. published a validation research investigating the psychometric properties of the Chinese version of the Body Shape Questionnaire (BSQ). Based on the participation of a large number of Chinese university students (*N* = 858), who presented at screening with an Eating Disorder Examination–Questionnaire Short (EDE-QS) score ≥15, the Chinese version of BSQ proved high internal consistency, statistically significant properties of differentiation between high-score and low-score group, good convergence validity and composite reliability. The importance of this study derives primarily from the significant relationships that could be established between body shape concerns and ED symptoms, making this a valuable tool for the assessment of ED in the Chinese population.

The cross-sectional study conducted by Alshahrani explored the very interesting phenomenon of food neophobia (FN) among a large sample of university students in Saudi Arabia (*N* = 480). Using an internet-based questionnaire, the prevalence of FN was 49.6%, and the most relevant protective factor for this condition was the will to engage in regular physical exercise. On the other hand, the study showed that food allergies, disordered attitudes toward eating, preference for fish and seafood, milk and dairy products, chocolate and candies, snacks, chips, nuts, and the use of dietary supplements were associated with a higher risk of FN. From a public health perspective, this study reflects the importance of screening for FN in adolescents and young adults, and for studies that further investigate the relationship between this condition and nutrition literacy and ED.

The retrospective cohort study conducted by Monaco et al. was focused on the investigation of the COVID-19 pandemic's influence on EDS in an Italian residency center, and explored thoroughly the presentations and severity of EDs in 162 patients admitted to the previously mentioned center, between December 2018 and December 2023. The study highlighted the need to construct multidisciplinary care models designed for EDs in the context of global crises, like a pandemic, especially for young adults, who presented with high rates of comorbidity and high rates of hospital readmissions.

Staśkiewicz-Bartecka et al. conducted a comparative cross-sectional study on orthorexia nervosa (OR) in relation to nutritional knowledge and insulin resistance (IR). The importance of the study, which included 133 female participants from a primary care clinic in Poland, derives from the need to increase awareness regarding the risk of ON onset in women with IR who also have a high level of dietary knowledge, due to their efforts to adhere to overly restrictive and rigid dietary recommendations.

Young reviewed the relation between ovarian hormones and EDs, especially anorexia nervosa (AN) and bulimia nervosa (BN), through the possible mediation of genetic pathways. This review supports the role of estrogens as contributors to the symptoms of AN, and incites the researchers to look at the anti-estrogenic drugs as potential therapeutic agents for this ED.

## 3 Conclusions

This collection is an argument for (1) considering EDs a global health problem, (1) developing public health policies aimed at screening the vulnerable populations, mainly adolescents and young adults, and (3) designing new and efficient therapeutic interventions. Research focused on the risk factors, pathogenic pathways, screening and monitoring psychometric tools focused on EDs, detection of comorbid disorders, and the exploration of the COVID-19 pandemic impact on the prevalence of EDs are the core domains that were explored in this topic. We consider that further exploration of the complex interplay between social, psychological, and biological factors in the onset and maintenance of EDs is warranted based on the currently available data. Other areas of interest in this field for future research could include prevention policies for EDs in patients with psychiatric or medical disorders, exploration of the role of public health in addressing risk factors and comorbidities of EDs, especially for obesity, ultra-processed food addiction, binge eating disorders, and AN, as well as the analysis of EDs in relation to changes in the ideal self-image in the post-modernistic society.
